# Factors Predicting Severe Dengue in Patients with Dengue Fever

**DOI:** 10.4084/MJHID.2013.014

**Published:** 2013-02-16

**Authors:** Muhammad Imran Hasan Khan, Eram Anwar, Adnan Agha, Noha Saleh Mohamed Hassanien, Ehsan Ullah, Imran Ali Syed, Arsalan Raja

**Affiliations:** 1 Medical Unit I, Lahore General Hospital, Lahore, Pakistan; 2 Armed Forces Hospital Southern Region, Khamis Mushyt, Saudi Arabia; 3 Lecturer of Biostatistics, High Institute of Public Health, Alexandria University, Egypt

## Abstract

**Introduction:**

Dengue virus (DENV) affects over half the world’s population in 112 countries, and dengue fever (DF) is the second largest arthropod borne infectious global hazard after malaria with complications like Dengue Hemorrhagic Fever (DHF) and Dengue Shock Syndrome (DSS), accounting for significant morbidity and mortality world-over. Pakistan is significantly affected with DENV infection and to-date no study identifying risk factors associated with development of severe complications of DF has been done.

**Methods:**

997 confirmed cases of DF were collected from a tertiary care hospital in Lahore, Pakistan and their clinical and biochemical data were collected. Univariate, multivariate and logistics regression analysis was performed to identify risk factors associated with development of DHF and DSS.

**Results:**

Bleeding OR 70.7 (CI 38.4–129.9), deranged liver function test OR 1.9 (CI 0.97–0.99), presence of urinary red blood cells OR 1.4 (95%CI 0.179–0.900) and presence of urinary protein OR 1.1 (95%CI 0.191–0.974) were related to development of DHF and DSS.

**Discussion:**

Severe Dengue, like DHF and DSS can be predicted by the presence of clinical and biochemical factors like signs of bleeding, deranged liver function test, presence of urinary red blood cells and urinary protein; so that the patients at high risk for complication be identified early and started on treatment timely.

**Conclusion:**

Predictors of severe dengue are identified in this study but further large scale multi-centered studies are needed for better interpretation.

## Introduction

Infection with the Dengue virus (DENV) is increasingly recognized as an important arthropod-borne viral infection infecting about 2.5 billion people worldwide; being endemic to over a 100 countries with over 975 million belonging to tropical and sub-tropical countries in Southeast Asia, the Pacific and the America.^1^–^3^ Dengue virus is a single stranded RNA virus which was first isolated from Japan in 1942 by Hotta;^4^ while belonging to Flaviviridae family it is transmitted to humans by infective female of Aedes genus, principally by Aedes aegypti, Aedes albopictus mosquito, Aedes polynesiensis and several species of the Aedes scutellaris complex.^5^ There are 4 antigenically distinct serotypes, i.e. DENV-1, DENV-2, DENV-3 and DENV-4, which are evolved from a common ancestor.^6^,^7^

Dengue viral infection can either cause dengue fever (DF), dengue hemorrhagic fever (DHF) or dengue shock syndrome (DSS). The classical dengue fever is mild, febrile illness which usually results after primary infection with dengue virus which is cleared in approximately seven days by a complex immune response.^8^ Secondary infection or infection with a different serotype can show severe outcomes characterized by high grade fever, increased vascular permeability, plasma leakage, hemorrhagic manifestations and thrombocytopenia due to antibody dependent enhancement and can lead to severe dengue, either DHF or DSS which can be life-threatening.^9^ Dengue fever has been described as early as 10^th^ century in Chinese illustrations but the first detailed description of dengue shock syndrome was accounted by Benjamin Rush in 1780.^10^ Dengue infection was first documented in Pakistan in year 1982 from Punjab in which 12 patients out of total 174 were found positive for dengue virus; all these samples were collected in 1968 and 1978.^11^ All four virus serotypes cause similar illness, but severe and fatal hemorrhagic disease is more often associated with DENV2 and DENV3 infections and both of them are the most commonly isolated genotypes in the Indo-Pak subcontinent.^12^

Clinical trials show various factors associated with development of DHF including DENV serotype, of ≤ 75,000/mm^3^ and hematocrit value of 50%, or a rise of more than 22% from baseline hematocrit, high viral load, intense activation of the immune system, various host conditions such as extremes of age, genetics, nutritional status, coexisting conditions and WHO recommended clinical criteria of abdominal tenderness, hepatomegaly, lethargy, cold extremity, and bleeding, in addition to Caucasian race, and people with AB blood group.^13^,^14^ To date there has been no study published on factors predicting development of DHF in patients from Pakistan with DF. The idea behind this study is to observe the clinical and biochemical characteristics of the patients with DF admitted to a tertiary care hospital in Pakistan and see if any of these factors are associated with prediction of poor outcome in terms of severe dengue like DHF, DSS and/or any mortality.

## Objective

To assess the clinical and biochemical factors affecting the disease severity and mortality among patients with dengue fever in a tertiary care hospital in Pakistan.

Severity was defined as presence of complications of dengue fever in the form of Dengue Hemorrhagic Fever or Dengue Shock Syndrome; both also known as Severe Dengue.

## Patient and methods

The study was conducted during the Dengue Fever epidemic in a tertiary care hospital (Lahore General Hospital/Postgraduate Medical Institute) in Lahore, Pakistan from August 1, 2011 till the September 30, 2011. The inclusion criterion was the presence of dengue fever (DF) which defined as finding three out of ten classical symptoms as described by World Health Organization (WHO) criteria and confirmed on the basis of presence dengue antibodies in a person from an endemic area.^9^ The widely used 1997 classification divided dengue into DF without any complications and dengue hemorrhagic fever DHF with further grades. Grade I and II being generally constituting DHF with grade I being presence only of easy bruising with confirmed dengue and grade II being presence of spontaneous bleeding into the skin and elsewhere; grade III and grade IV represented DSS with grade III being presence of clinical evidence of shock, and grade IV being undetectable blood pressure and pulse.^9^ The 2009 classification simply places all patients with confirmed DF without any complications in uncomplicated dengue fever group while all confirmed DF cases with presence of severe organ impairment, plasma leakage or hemorrhage were included in severe dengue group, meaning DHF and DSS are both considered as severe dengue.^15^

The patients who were found to be negative for DF antibodies by Enzyme-linked immunosorbent assay (ELISA) or who had another cause identified for their symptoms like malaria, sepsis or infection elsewhere were excluded from the study. The data regarding physical characteristics including signs and symptoms, biochemical data, complications in the form of DHF and DSS and outcome in terms of numbers of days admitted and mortality if any, were recorded on a specially designed proforma. Data were then entered and analyzed using SPSS (statistical package for social sciences) for windows version 16. Data were tested for normality using Kolmogrov and Smirnove test. Categorical variables were presented as number and percentage while numerical variables were presented as mean (standard deviation) if normally distributed or median (range) if not normally distributed. Univariate and multivariate statistical analysis were undertaken successively.

A univariate analysis was carried out using ANOVA test or Kruskal Wallis test for comparison between continuous variables, whereas the χ2 test or Monte Carlo exact test was used to assess differences between Categorical variables. Multivariate analysis was carried out by using Multinomial logistic regression analysis (MLR) where all the significant variables in the univariate analysis were included in multivariate analysis to adjust for confounders. This model was used to assess the association between the predictors (demographic, clinical and laboratory variables) and multinomial outcome of each case as DF, DHF or DSS (3 possible outcomes). DF was considered as the reference group. Categorical independent variables were included as “factors” while quantitative independent variables were included as “covariates”. Odds ratio (OR) with 95% confidence intervals (95%CI) were used to identify the likelihood of categorical variables predicting the outcome. A p-value of 0.05 or less was taken as significant.

## Results

Of the 1200 patients initially included in this study, 223 were excluded with other causes of febrile illness. Overall 977 were confirmed as dengue illness; including 686 cases with DF (70.2%), 280 cases with DHF (28.7%) and 11 cases with DSS (1.1%).

### Patient’s Demographics

Among 977 confirmed dengue illness, ages ranged from 5 to 87 years with a mean + SD age of 39.9 + 6.7 years and there was no significant difference between different diagnosis (DF, DHF and DSS) regarding age (see table 1 for details). The majority of cases were males (70%) which was a similar trend among patients of DF (70%) and DHF (74%) but the patients who developed DSS majority were females (64%) and this difference was significant (P <0.05). There was no significant difference between different disease classification (DF, DHF and DSS) in both percent of IgM positivity and outcome. DSS had much higher percent of IgG positivity (81.8%) compared with 71.4% in DHF and 59.5% in DF and this difference was significant. The majority of DSS cases (72.7%) required intervention compared to 25.4 % in DHF and 6.1% in DF and his difference was also significant.

### Clinical Manifestations

There was no significant difference between DF and complicated cases (DHF and DSS) in presentation with headache, high fever, vomiting, abdominal pain and ascites and pleural effusion (see table 2 for details). Complicated cases were significantly associated with higher percent of backache, bleeding and encephalitis (with the highest percent among DSS), while rash is much significantly lower in DSS.

### Laboratory tests

It was seen that majority of patients with urinary proteins (URP) and Urinary Red blood cells (URBC) (72.7%) develop shock (see table 3 for details). Platelet count was significantly lower in both DHF and DSS than DF also in it was significantly lower in DSS than DHF. Patients with high Aspartate aminotransferase (AST) and Alanine aminotransferase (ALT) levels also tend to develop shock.

### Multivariate Analysis

A multivariate analysis was carried out by using Multinomial logistic regression analysis (MLR) where all the significant variables in the univariate analysis were included in multivariate analysis to adjust for confounders. This model was used to assess the association between the predictors (demographic, clinical and laboratory variables) and multinomial outcome of each case as DF, DHF or DSS (3 possible outcomes). DF was considered as the reference group. Categorical independent variables were included as “factors” while quantitative independent variables were included as “covariates”. The interpretation of MRL results was as following:

### Overall model evaluation

The relationship between the dependent and independent variables was evaluated; the correlation was based on the statistical significance of the chi-square model. In this analysis, the distribution reveals that the probability of the chi-square model (54.3) was 0.003; that suggests a statistically significant relationship between the independent variable and the dependent variable, being the level of significance <0.05 (i.e. p<0.05).

### Strength of Multinomial Logistic Regression Relationship

Once the relationship is established, the next important thing to do is to test the strength of multinomial logistic regression relationship.

1. Using Cox & Snell R Square and the Nagelkerke R square value, they provide an indication of the amount of variation in the dependent variable, these are described as pseudo R square, this model revealed that the values are 0.181 and 0.322 respectively, suggesting that between 18.1% percent and 32.2% percent of the variability is explained by this set of variables used in the model.
2. The proportional by chance accuracy rate was computed by calculating the proportion of cases for each group based on the number of cases in each group and then squaring and summing the proportion of cases in each group (0..702^2^ + 0.287^2^ + 0.011^2^ = 0.575). The proportional by chance accuracy criteria however was 71.9% (1.25 × 57.5% = 71.9%). The classification accuracy rate was 86.1% which was greater than the proportional by chance accuracy criteria of 71.9%, suggesting that the model was useful.

### Statistical tests of individual predictors

These were tested using Wald test which evaluates whether or not the independent variable is statistically significant in differentiating between two groups in each of embedded binary logistic comparisons.

Multivariate analysis showed that presence of bleeding, serum ALT, URP and URBC were significantly associated with DHF. From multinomial logistic regression model, bleeding had an OR of developing DHF 70.7 times that of DF, a unit increase in ALT had an OR of developing DHF 1.9 times that of DF, URP had an OR of developing DHF 1.1 times that of DF and URBC had an OR of developing DHF 1.4 times that of DF (See table 4 for details).

While on univariate analysis DSS was more common in females than males with a significant P-value of 0.02 as well as high titers of IgG were associated with development of DSS with a significant P-value of 0.001; but on assessing predictors of DSS on multivariate analysis, bleeding was a significant predictor of DSS in addition to serum ALT and AST. Multinomial logistic regression model showed that bleeding had an OR of developing DSS 608.2 times that of DF; while each unit increase in serum ALT and AST had an OR of developing DSS 1.04 and 1.05 respectively times that of DF.

## Discussion

Dengue infection has emerged as a major health concern in Southeast Asia, the pacific and America. In Pakistan as well Dengue virus has become a serious issue and it has caused many endemics starting from 1994 to 2011. In 1985, a research conducted to study the prevalence of dengue virus infection showed that about 60% of the Pakistanis were haemagglutination inhibition (HI) antibody positive for West Nile, Japanese encephalitis and DENV-2 Flaviviruses, which rapidly increased from July to October in patients ranging from 6 to 20 year age.^16^ In 1994, first outbreak of DHF was reported in Pakistan in which 15 out of 16 patients had dengue IgM while three out of ten patients of dengue virus were infected with DEN-1 and DEN-2.^17^ In 2005, outbreak of DHF in Karachi, DEN-3 was reported among the few tested patients while DEN-2 and DEN-3 were found to be co-circulated during 2006 outbreak in Karachi.^18^,^19^ In 2008, a dengue outbreak was reported in Lahore infecting a large number of citizens of Lahore and were found to have DEN-4, DEN-2 and DEN-3 infection.^20^ In our study however, we could not do analysis for further serotypes and we only performed IgG and IgM antibodies via ELISA method.

Recent studies have shown that in addition to recommended WHO criteria, AB blood type, Caucasian race; new clinical and biochemical markers like clinical bleeding, high serum urea, low serum protein, low lymphocyte proportion, viral load assessment, viral serotype testing, cytokine, elastase, hyaluronan, soluble thrombomodulin, and nitric oxide level, and circulating endothelial cell detection test are associated with development of complications like DHF and DSS.^21^,^22^ Our study showed that severity of DF in the form of risk of developing DSS can be predicted by presence of high titers of IgG, significant P-value of 0.001; signs of bleeding, p-value < 0.0001; and raised serum ALT and AST, p-value of 0.01 and 0.004 respectively. While the risk of developing DHF in patient with DF can be predicted by presence of bleeding, URP and URBC and raised serum ALT. To-date no study from Pakistan has been published assessing the risk factors associated with developing complications in patients with DF.

Dengue virus circulates in Pakistan throughout the year with a peak incidence in the post monsoon period which is made worse by floods and as there is no licensed vaccine or antiviral compounds available against dengue virus, prevention and early identification remains the keystone of treatment. Recently there have been studies on ovicidal and adulticidal activities of plant leaf extracts against mosquito vector which appears to show promise in the future for widespread use as an ideal eco-friendly insecticide for the control of mosquitoes that spread malaria, yellow fever, filariasis and other arboviruses.^23^

We need to identify patients at risk of developing severe complications and treat them promptly. Until we come up with an antiviral compounds that can target all four serotypes of dengue with same efficiency, recognizing factors that can help predict well in advance the severe form of DF, like DHF and DSS, could focus treatment in these group of patients. Our study showed increased liver function tests in serum, OR 1.9 (95%CI 0.97–0.99); presence of URBC, OR 1.4 (95%CI 0.179–0.900); and presence of URP, OR 1.1 (95%CI 0.191–0.974) can help predict patients at high risk for developing severe DF, both DHF and DSS. The most significant factor in identifying severe DF is the development bleeding (internal or external) in patients with DF which had OR 70.7 (95%CI 38.4–129.9), making them susceptible to develop DHF; while the risk for progressing to DSS in them is even more, OR 608.2 (95%CI 33.9–108.9). More studies that can help add these factors as part of the warning signs for prediction of severe DF.

## Conclusion

Dengue infection has emerged as a major health concern in Southeast Asia especially Pakistan and till a specific vaccine or an antiviral agent become available, we have to rely on early identification of risk factors associated with developing complications of DF; just like the warning features specified by WHO as well as the ones identified in this study. Our study attempts to identify clinical and biochemical factors like signs of bleeding, deranged liver function test, presence of urinary red blood cells and urinary protein as possible predictors of severe DF so that a proactive approach is taken to treat these patients. However more large scale multi-centered trials are needed to confirm the findings in this study and to may be add these predictors later-on as a part of the warning signs for development of severe DF.

## Figures and Tables

**Table 1:** Characteristics of patients with dengue fever according to final diagnosis

Variables	Dengue fever (n = 686)	Dengue hemorrhagic Fever (n = 280)	Dengue shock syndrome (n = 11)	Total (n = 977)	Test of significance

**Age (mean± SD)**	39.7 ±16.7	39.9 ± 17.3	47.5 ±18.9	39.9 ±16.7	F =1.2, P = 0.303

**Male, no. (%)**	480 (70)	207(74)	4(36)	691(70)	X^2^ =7.8, P = 0.020*

**Outcome, no. (%)**					
➢ **Recovery**	660(96.6)	276(98.9)	11(100)	948 (97.3)	MCE, P = 0.053
➢ **LAMA**	23(3.4)	2(0.7)	0	27 (2.6)	
➢ **Death**	0	1(0.4)	0	1 (0.1)	

**Intervention, no. (%)**	42 (6.1)	71 (25.4)	8 (72.7)	121 (12.4)	X^2^ =105.1, P = 0.000*

**Positive IgM, No. (%)**	579(84.4)	226(80.7)	10(90.9)	518 (83.4)	X^2^ =2.4, P = 0.300

**Positive IgG, No. (%)**	408(59.5)	200(71.4)	9(81.8)	617 (63.2)	X^2^ =13.8, P = 0.001*

F (ANOVA test)- X^2^ (chi-square test)- MCE (Monte Carlo exact test)

SD= Standard Deviation; LAMA= Left Against Medical Advice

* Significant (P < 0.05)

**Table 2:** Comparison of symptoms among patients with Dengue Fever (DF), Dengue Hemorrhagic Fever (DHF) and Dengue Shock Syndrome (DSS)

Presence of symptom	DF (n = 686)	DHF (n = 280)	DSS (n = 11)	Total (n = 977)	Test of significance
**Headache, no. (%)**	684 (99.7)	274 (97.9)	11 (100)	969 (99.2)	MCE, P =0.095
**High fever, no. (%)**	684 (99.7)	280 (100)	11 (100)	975 (99.8)	MCE, P =0.592
**Backache, no. (%)**	157 (22.9)	87(31.1)	3(27.3)	247(25.3)	X^2^ =7.1, P = 0.029*
**Encephalitis, no. (%)**	4(0.6)	9(3.2)	6(54.5)	19 (1.9)	X^2^ =168, P = 0.000*
**Bleeding, no. (%)**	14(2.0)	275(98.6)	10(90.9)	299 (30.6)	X^2^ =88.6, P = 0.000*
**Vomiting, no. (%)**	417 (60.9)	173 (61.8)	8 (72.7)	598 (61.3)	X^2^ =.68, P = 0.710
**Rash, no. (%)**	288 (42.2)	131 (47.1)	1 (9.1)	420 (43.2)	X^2^ =7.3, P = 0.027*
**Abdominal pain, no. (%)**	363 (53.0)	152 (54.3)	7 (63.6)	522 (53.5)	X^2^ =.59, P = 0.743
**Ascites, no. (%)**	185 (27.0)	84 (30.0)	4 (36.4)	273 (27.9)	X^2^ =1.3, P = 0.522
**Pleural effusion, no. (%)**	146 (21.5)	66 (23.7)	4 (36.4)	216 (22.3)	X^2^ =1.8, P = 0.402

X^2^ (chi-square test)- MCE (Monte Carlo exact test)

* Significant (P < 0.05)

**Table 3:** Comparison of Laboratory tests among patients with Dengue Fever (DF), Dengue Hemorrhagic Fever (DHF) and Dengue Shock Syndrome (DSS)

	DF (n = 686)	DHF (n = 280)	DSS (n = 11)	Total (n = 977)	Test of significance
**Presence of Urinary Proteins, no. (%)**	42 (6.1)	64 (22.9)	8 (72.7)	114 (11.7)	X2 =94.3, P = 0.000*
**Presence of Urinary Red blood cells, no. (%)**	42 (6.1)	71 (25.4)	8 (72.7)	121 (12.4)	X2 =105.1, P = 0.000*
**Platelets (x 109/L) at admission, Median (range)**	70500 (5800–1410000)	61000 (5800–429000)	35000 (15000–114000)	66000 (5800–1410000)	K= 20.5, P = 0.000*
**Platelets (x 109/L) during admission, Median (range)**	65000 (1800–185000)	50000 (5000–185000)	26000 (9000–34000)	60000 (1800–185000)	K=36.6, P = 0.000*
**Platelets (x 109/L) on discharge, Median ( range)**	56000 (7000–1110000)	45000 (7000–890000)	18000 (10000–28000)	51000 (7000–1110000)	K=45.7, P = 0.000*
**Asparate aminotranferase (IU/ml), Median (range)**	58 (11–189)	56 (23–186)	133 (9–140)	58 (9–189)	K=19.3, P = 0.000*
**Alanine aminotranferase (IU/ml), Median (range)**	56 (14–184)	56 (29–186)	125 (98–182)	56 (14–186)	K=32.7, P = 0.000*

X2 (chi-square test)- K (Kruskal Wallis Test)

* Significant (P < 0.05)

**Table 4:** Multinomial logistic regression analysis of factors associated with dengue complications (Dengue Hemorrhagic Fever and Dengue Shock Syndrome)

Variable	Dengue Hemorrhagic Fever (n = 280)	Dengue Shock Syndrome (n = 11)
	P-value OR (95% CI)	
**Presence of bleeding**	0.000 70.7 (38.4–129.9)	0.000 608.2 (33.9–1008.9)
**Alanine aminotranferase level**	0.001 1.9 (0.97–2.99)	0.004 1.04 (1.015–1.080)
**Aspartate aminotransferase level**	NS	0.010 1.05 (1.012–1.088)
**Urinary Proteins**	0.043 1.1 (0.191–1.974)	NS
**Urinary Red blood cells**	0.027 1.4 **(**0.179–1.900**)**	NS
**Platelet count on admsission less than 50,000 × 10^9^/L**	NS	0.014 0.16 (0.13–0.19)

N.B. Results of significant predictors for either Dengue Hemorrhagic Fever or Dengue Shock Syndrome are included in the table. NS = Not significant
